# Supplementary material for: Hypoxia-induced SETX links replication stress with the unfolded protein response
Source: Nat Commun. 2021 Jun 17;12:3686. doi: 10.1038/s41467-021-24066-z (PMC8211819; doi:10.1038/s41467-021-24066-z)
Supplement: Supplementary file 1 — Supplementary Information [file 41467_2021_24066_MOESM1_ESM.pdf]

Hypoxia-induced SETX links replication stress with the unfolded protein response

Shaliny Ramachandran[1+], Tiffany S. Ma[1+], Jon Griffin[3,4], Natalie Ng[1], Iosifina P. Foskolou[1], Ming-Shih Hwang[1], Pedro Victori[1], Wei-Chen Cheng[1], Francesca M. Buffa[1], Katarzyna B. Leszczynska[1], Sherif F. El-Khamisy[3,5], Natalia Gromak[2], and Ester M. Hammond[1$]

[1]Oxford Institute for Radiation Oncology, Department of Oncology, University of Oxford, Oxford, UK. [2]Sir William Dunn School of Pathology, University of Oxford, Oxford, UK [3]Healthy Lifespan and Neuroscience Institute, Department of Molecular Biology and Biotechnology, Firth Court, University of Sheffield, Sheffield, UK, [4] Department of Histopathology, Sheffield Teaching Hospitals NHS Foundation Trust, UK. [5]Institute of Cancer Therapeutics, University of Bradford, UK.

Contents

SI Tables	Page	2
SI Methods	Page	3
SI Figures	Page	4
SI References	Page	13

Table S1 – Primer sequences used

GENE	FORWARD	REVERSE	SOURCE
18S	GCCCGAAGCGTTTACTTTGA	TCCATTATTCCTAGCTGCGGTATC	Kreig et al. *MCB* 2010 [1]
SETX	CTTCATCCTCGGACATTTGAG	TTAATAATGGCACCACGCTTC	Skourti-Stathaki et al. *Mol. Cell*. 2011 [2]
RNase H1	CCTGTACTTACTGGTGTGGA	CCGTGTGAAAGACGCATCTG	Wu et al. *PLOS One*. 2013 [3]
RNase H2B	GCATCTTGTTGCTGAAACTTC	TCCTTGTCAGTGGAAGCTTG	Pizzi et al. *Hum. Mol. Genet.* 2014 [4]
PIF1	CGAGCCTAGCACAGAAGCC	CCCAGGATTCGCTTTAGCAG	Primer Bank [5]
AQR	GGCGGCATTAGCTGAAACTG	CAGCTGTAAGCCCATCCACA	Primer Blast [6]
DHX9	GCCAATTTCTGGCCAAAGCA	CGAGGCTCAATGGGGAGTTT	Primer Blast [6]
RTEL1	GGACTTGGTCAAGAGCGGAA	AGTCAGGTCAAAGGATGCCG	Primer Blast [6]
TCF19	GGGCGGTGATCTCTACACCT	ACCTCTTGGGAGTCGGACAT	Primer Blast [6]
GHITM	CGTCCTTTCGATGTTGCGTC	TTCTTCACAACAGGGGAGGC	Primer Blast [6]
FAM222A	CCAAGGAGGGCTTCGTCAG	CAGCTCCAGGCTCTTGCTC	Primer Blast [6]
ZNF367	TTTCCACGGGAGAAATCGCT	GGTGTGAAGACGCTGATGTG	Primer Blast [6]
MMP1	CATGCTTTTCAACCAGGCCC	GGGTACATCAAAGCCCCGAT	Primer Blast [6]
JUN	GTGCCGAAAAAGGAAGCTGG	CTGCGTTAGCATGAGTTGGC	Primer Blast [6]
TM4SF	TACACCTTTGCCAGCACTGA	TTGCTGTTGGTGAGAGCAGC	Primer Blast [6]
GDF15	GTTGCACTCCGAAGACTCCA	GAGAGATACGCAGGTGCAGG	Primer Blast [6]
CHOP	GGAGCATCAGTCCCCCACTT	TGTGGGATTGAGGGTCACA	Rouschop et al. *JCI.* 2010 [7]
VEGF	CTACCTCCACCATGCCAAGT	CTCGATTGGATGGCAGTAGC	Dobrynin et al. *Sci. Rep.* 2017 [8]
ATF4	TGACCTGGAAACCATGCCAG	AATGATCTGGAGTGGAGGAC	Zong et al. *BBA.* 2012 [9]
PHLDA3	GCGCCACATCTACTTCACG	CACAAGCCAGAGGGAACAAC	Leszczynska et al. *JCI.* 2015 [10]

Table S2 - siRNA sequences used

siRNA	Sequence
SETX-A	GCACGUCAGUCAUGCGUAA,<br>GCAAUAAGCUCAUCCUAGU,<br>GCUCAACUCUCCAAAUAGA,<br>UAGCACAGGUUGUUAAUCA
SETX-B	CCAAUUGCUCCUUUCAGGUGUUUGA
HIF1α	CUGAUGACCAGCAACUUGAdTdT
p53	GUAAUCUACUGGGACGGAA
PERK	CCAAUGGGAUAGUGACGAA,<br>GGUAGGAUCUGAUGAAUUU,<br>GCAAUUAGCCUUAAGUUGU,<br>AAAUUUGGCUGAAAGAUGA
ATF4	CCACGUUGGAUGACACUUGdTdT

SI Methods

Visualising RNA/DNA hybrids by slot blot assay

The slot blot assay was adapted from a previously described method [11]. Cells were harvested in lysis buffer (100 mM Tris-HCl pH 8.5, 5 mM EDTA, 0.2% SDS, and 100 mM NaCl containing 0.5 mg/ml proteinase K) and incubated in a 55°C heated shaker overnight. Genomic DNA was extracted with isopropanol, washed in 70% ethanol, air-dried and re-suspended in TE buffer. 50 µg of genomic DNA was treated with 2 µL of RNase A (Fermentas) for 2 hours, and using a slot blot apparatus (Bio-Rad), equal amounts of samples were blotted onto a positively charged nylon transfer membrane (GE Healthcare). Where indicated, samples were treated with RNase H1 (NEB) overnight. The ssDNA blot was denatured (0.4 M NaOH and 0.6 M NaCl) and neutralised (1.5 M NaCl and 0.5 M Tris pH 7.4). Blots were UV crosslinked (0.12 J/m^2), blocked then incubated with S9.6 (1:1000, Kerafast) or ssDNA (1:5000, Millipore) antibodies and imaged using the Odyssey Infrared system. Intensity of bands were quantified using ImageJ software. The images were converted to 8-bit format. Each band was individually selected and circumscribed with the rectangular ROI selection and "Gels" function, followed by quantification of peak integral area from each band's corresponding histogram.

RNA-sequencing Enrichment analysis

Several tools were used for the gathering of data on the differentially expressed genes. For G-quadruplex sequences, QuadBase was used [12]. We relied on BiomaRt for a series of measurements, namely gene length, number of exons and GC percent [13].

Supplementary Figure 1

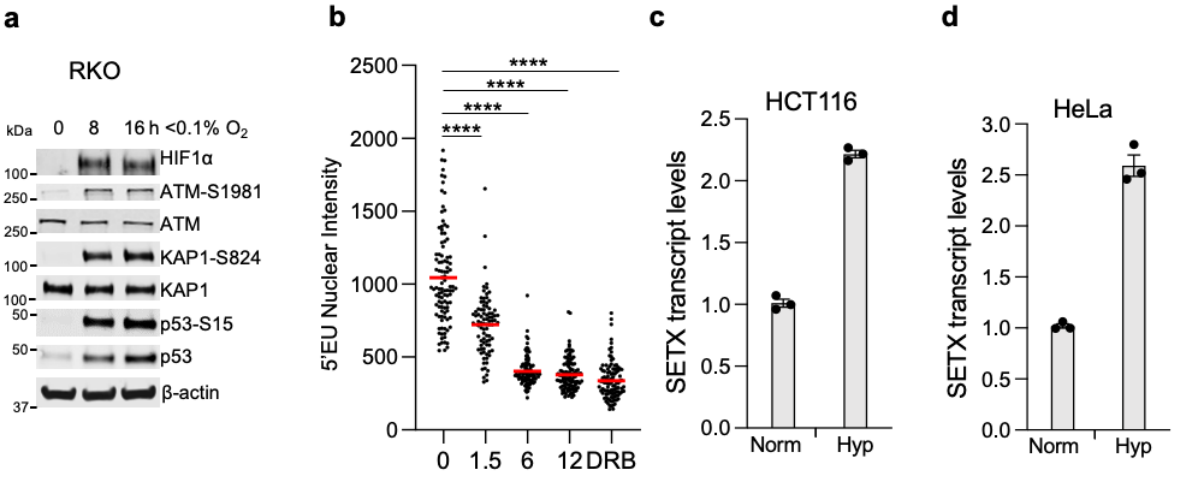

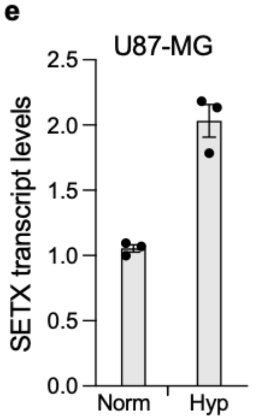

f

Correlation of expression of SETX and hypoxia signature		
**TCGA datasets:**	**Spearman:**	***P*:**
Lung squamous cell carcinoma (n= 501)	-0.31	<0.001
Lung adenocarcinoma (n= 517)	-0.38	<0.0001
Colorectal (n=382)	-0.37	<0.0001

Supplementary Figure 1. **SETX is induced in response to hypoxia**
a RKO cells were exposed to <0.1% O_2 for 0, 8 and 16 hours followed by western blotting with the indicated antibodies, n=3. **b** A549 cells were exposed to <0.1% O_2 for 0, 1.5, 6 and 12 hours with 5'EU (5-ethynyluridine) (0.5 mM) was added for the final hour. Representative of 2 independent experiments (n=2) where each dot represents a cell and a minimum of 100 cells were imaged per treatment. One-way ANOVA Tukey's multiple comparisons test was used: ****=p<0.0001. **c** HCT116 cells were exposed to <0.1% O_2 for 6 hours followed by RT-qPCR for the relative mRNA levels of SETX determined by RT-qPCR, Representative of 2 independent experiments (n=2). **d** HeLa cells were exposed to <0.1% O_2 for 6 hours followed by RT-qPCR for the relative mRNA levels of SETX determined by RT-qPCR, Representative of 2 independent experiments (n=2). E. U87-MG cells were exposed to <0.1% O_2 for 6 hours followed by RT-qPCR for the relative mRNA levels of SETX determined by RT-qPCR. Mean ± standard error of the mean (SEM) are displayed (**c**, **d** and **e**), Representative of 2 independent experiments (n=2). **f** Expression analysis of SETX in The Cancer Genome Atlas (TCGA) datasets. RNA-sequencing data (RNA Seq V2 RSEM) for 382 colorectal adenocarcinoma tumours, 517 lung adenocarcinoma tumours and 501 lung squamous cell carcinoma tumours were downloaded from the TCGA project. A correlation of SETX was determined against hypoxia metagene signature [14]. Correlations and statistical significance were determined by calculating Spearman's rho rank correlation coefficient (r) and two-tailed *P* value using Hmisc package in RStudio: Lung squamous cell carcinoma (p<0.001), Lung adenocarcinoma (p<0.0001), Colorectal (p<0.0001).

Supplementary Figure 2
legend appears on following page

a

term ID	term name
GO:0034660	ncRNA metabolic process
GO:0016072	rRNA metabolic process
GO:0022613	ribonucleoprotein complex biogenesis
GO:0042254	ribosome biogenesis
GO:0034470	ncRNA processing
GO:0006364	rRNA processing
GO:0030490	maturation of SSU-rRNA
GO:0030684	preribosome
GO:0032040	small-subunit processome
GO:0005730	nucleolus
GO:0044452	nucleolar part
GO:0030515	snoRNA binding
CORUM:5101	CyclinD3-CDK4-CDK6-p21 complex

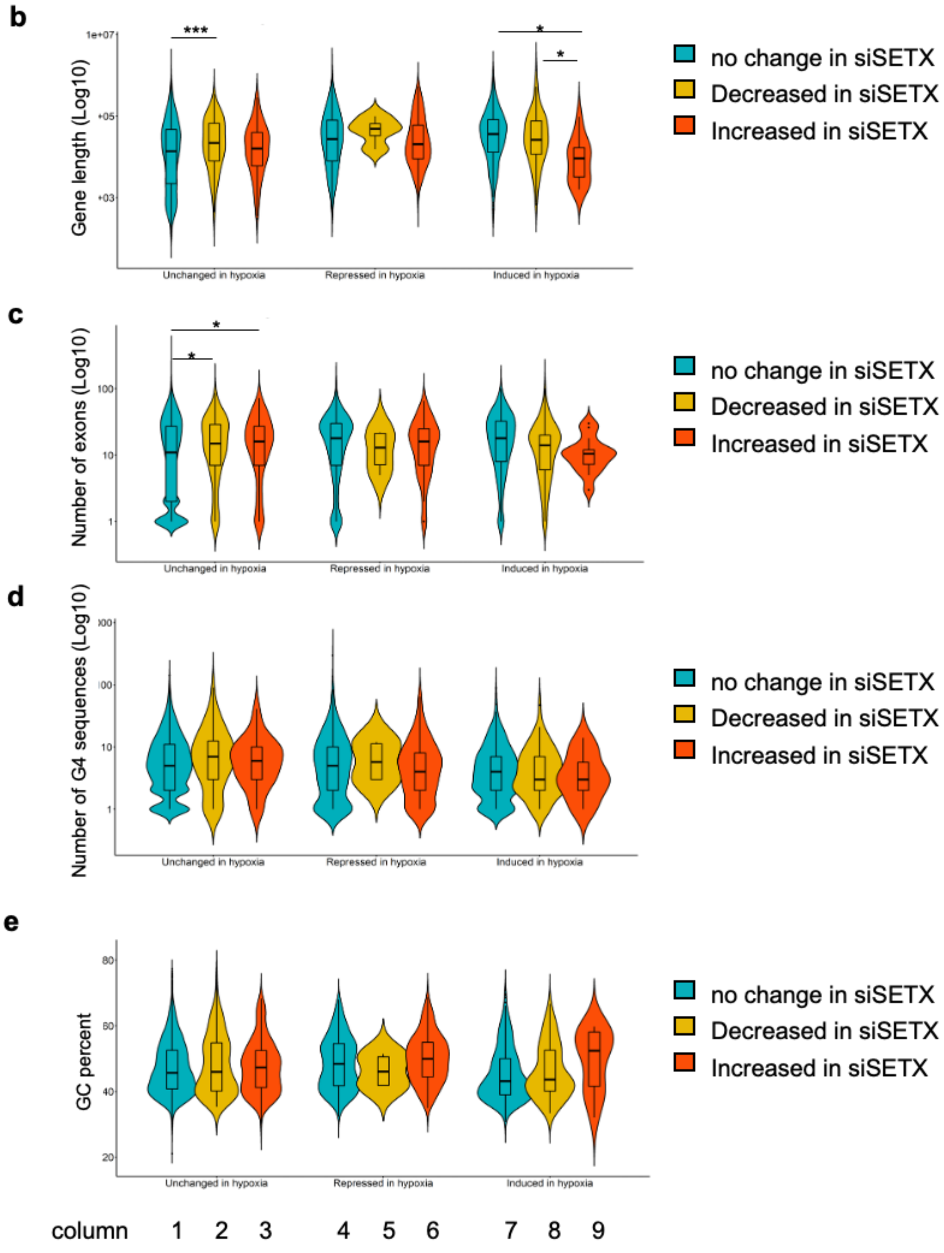

Supplementary Figure 2. **Analysis of genes changing in a SETX-dependent manner**

a Gene ontology analysis found that a number of genes (69/341) upregulated in SETX depleted hypoxic cells were involved in rRNA processing, nucleolus and ribosome biogenesis. **b** All genes from the RNA-sequencing were grouped into 3 sections: (1) Genes that do not change in hypoxia (column 1-3), (2) genes that are repressed in hypoxia (column 4-6), and (3) genes that are induced in hypoxia (column 7-9). Within each of the 3 sections, the length of genes was compared between genes do not change expression in siSETX hypoxic cells (green) to genes that decrease in siSETX hypoxic cells (yellow) and to genes that increase in siSETX hypoxic cells (orange). RNA-seq was carried out in 3 biological replicates (n=3). ***=p=0.0004; genes induced in hypoxia and unchanged in siSETX versus induced hypoxia and increased in siSETX *=p=0.0100; genes induced in hypoxia and decreased in siSETX versus induced hypoxia and increased in siSETX *=p=0.0411. Total number of genes in **b** = 25330. **c** Same analysis as **b** comparing number of exons. Genes unchanged in hypoxia and unchanged in siSETX versus unchanged in hypoxia and decreased in siSETX *=p=0.0107; genes unchanged in hypoxia and unchanged in siSETX versus unchanged in hypoxia and increased in siSETX *=p=0.0158. Number of genes = 25330. **d** Same analysis as **b** comparing number of G4 sequences. Number of genes = 4669. **e** Same analysis as **b** comparing GC content. Number of genes = 25582.

(**b**-**e**) For box plots, centre line = median; upper and lower bounds of the whiskers = 1.5 times the interquartile range from the corresponding ‘hinge’ (upper or lower bound of the box); bounds of the box = lower hinge is the 25% quantile and upper hinge is the 75% quantile; bounds of the whiskers = extend from bounds of the box to the minima and maxima; data beyond the end of the whiskers are plotted individually. Statistical significance was determined using the two-sided Wilcoxon test, and p-values were adjusted with the Holm method.

Supplementary Figure 3

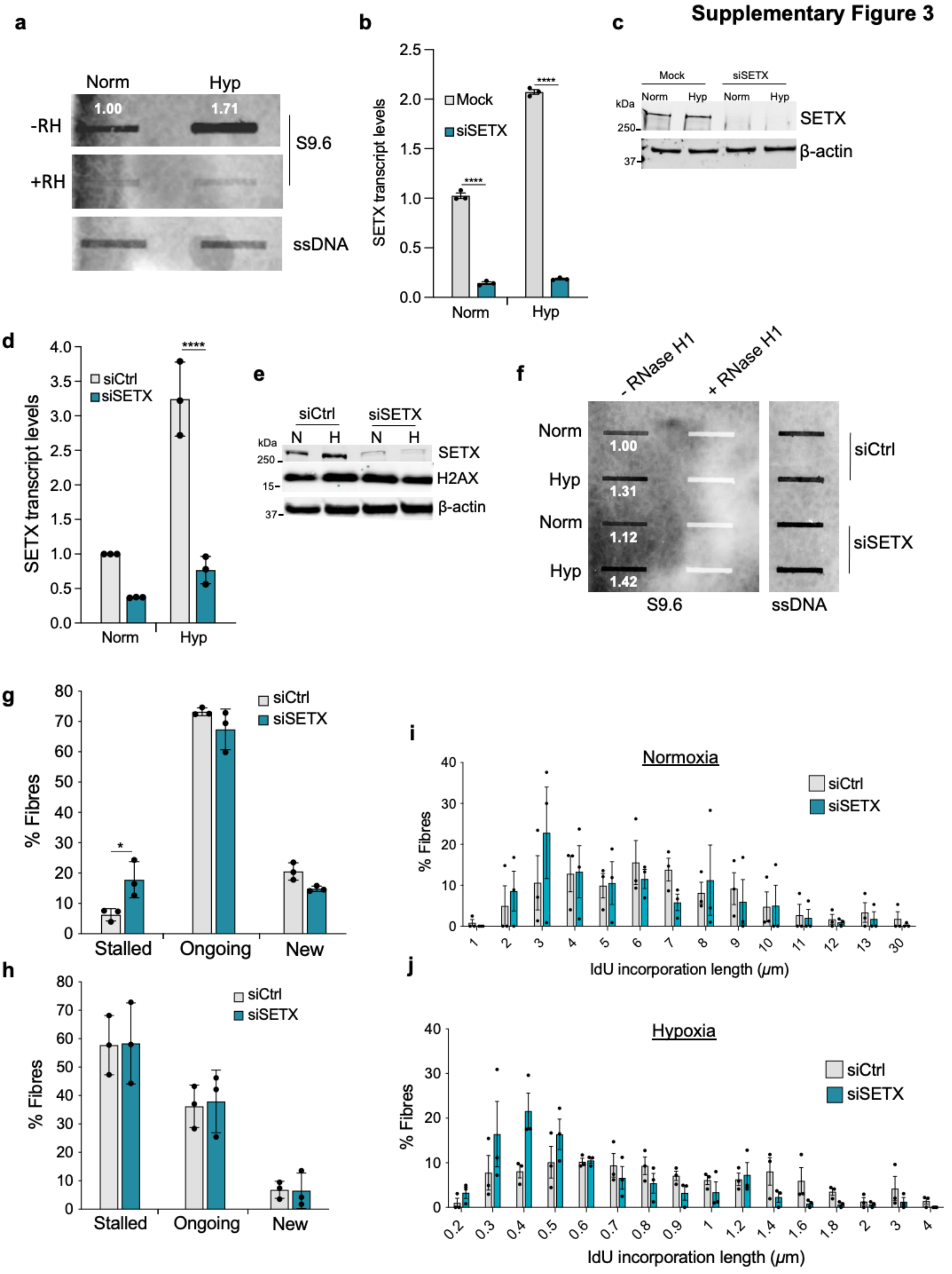

Supplementary Figure 3. **SETX depletion leads to increased R loops and replication stress in hypoxia**
a RKO cells were exposed to 21% or <0.1% O_2 for 6 hours. Genomic DNA was extracted, treated with or without RNase H (RH) and subjected to slot blot assays with the S9.6 antibody. ssDNA was used as the loading control. Numbers show quantification of S9.6 intensity fold change normalised to ssDNA, relative to untreated normoxic sample. **b** SETX transcript levels as determined by RT-qPCR in A549 cells with or without SETX siRNA after 16 h hypoxia (<0.1% O_2) treatment. To support the data shown in Figure 3A. Representative of 2 independent experiments (n=2), mean ± standard deviation (SD) are displayed. One-way ANOVA Tukey's multiple comparisons test was used: ****=p<0.0001. **c** Western blot analysis in A549 cells with or without SETX siRNA after 6 hours in normoxia or hypoxia (<0.1% O_2). To support the data shown in Figure 3A. **d** SETX transcript levels as determined by RT-qPCR in RKO cells with either siCtrl or SETX siRNA after 16 h hypoxia (<0.1% O_2) treatment. Data from 3 independent experiments (n=3), mean ± SD are displayed. One-way ANOVA Tukey's multiple comparisons test was used: ****=p<0.0001. **e** Western blot analysis in RKO cells with control siRNA or SETX siRNA after 6 hours in normoxia or hypoxia (<0.1% O_2). **f** RKO cells were transfected with control siRNA (siCtrl) or SETX siRNA and exposed to 21% or <0.1% O_2 for 6 hours. Genomic DNA was extracted, treated with or without RNase H and subjected to slot blot assays with the S9.6 antibody. ssDNA was used as the loading control. Numbers show quantification of S9.6 intensity fold change normalised to ssDNA, relative to untreated normoxic sample. Representative of 2 independent experiments. **g** RKO cells transfected with control siRNA (grey) or SETX siRNA (green), were subjected to DNA fibre assays at 21% O_2 (Normoxia) and the different types of replication structures were quantified. Mean ± SD are displayed. 2-tailed Student's t test was used: p=0.0338. **h** RKO cells transfected with control siRNA or SETX siRNA, were subjected to DNA fibre assays at <0.1% O_2 (Hypoxia) and the different types of replication structures were quantified. **i** Replication rate curves for RKO cells with control siRNA (siCtrl, grey) or SETX siRNA (green), as measured by IdU incorporation rates using immunofluorescence microscopy, in normoxia (21% O_2). Figure generated using the same data as Supplementary Fig. 3g. **j** Replication rate curves for RKO cells with control siRNA (siCtrl, grey) or SETX siRNA (green), as measured by IdU incorporation rates using immunofluorescence microscopy, after 6 hours of hypoxia (<0.1% O_2) treatment. Figure generated using the same data as Supplementary Fig. 3h.
(**a-j**) Data from three independent experiments (n=3), mean ± SEM are displayed unless otherwise indicated.

Supplementary Figure 4

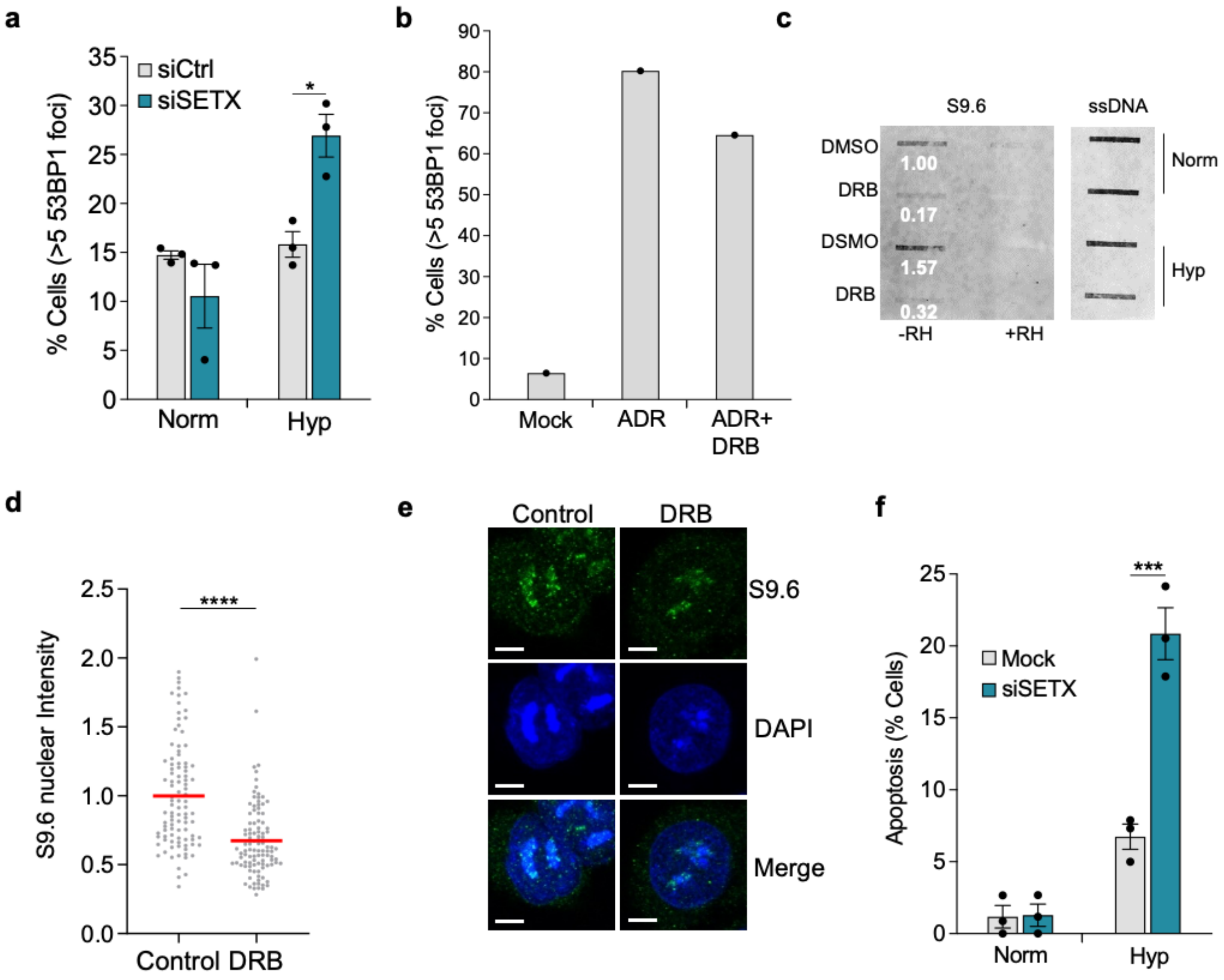

Supplementary Figure 4. **Loss of SETX in hypoxia increases DNA damage and apoptosis**

a Percentage of cells displaying 53BP1 foci (>5 per nucleus) in RKO cells with either control siRNA or SETX siRNA. Cells were exposed to 21% O_2 (Norm) or <0.1% O2 (Hyp) for 6 hours. Data from three independent experiments (n=3), mean ± SEM are displayed. One-way ANOVA Tukey's multiple comparisons test was used: *=p=0.0227. **b** Percentage of cells displaying 53BP1 foci (>5 per nucleus) in RKO cells exposed to mock treatment, Adriamycin (ADR, 2 µM) or pre-treatment with DRB (100 µM) followed by Adriamycin (2 µM) for 6 hours. Data from one independent experiment (n=1). **c** RKO cells were pre-treated with DMSO or DRB (100 µM) and exposed to 21% or <0.1% O_2 for 6 hours. Genomic DNA was extracted, treated with or without RNase H (RH) and subjected to slot blot assays with the S9.6 antibody. ssDNA was used as the loading control. Numbers below bands show the S9.6 signal normalised to ssDNA, relative to normoxic untreated control. n=1 **d** A549 control cells or cells treated with DRB (100 µM) for 6 hours, were fixed and stained with the S9.6 antibody and DAPI. Representative of 2 independent experiments (n=2) where each dot represents a cell and a minimum of 100 cells were imaged per treatment. The two-tailed Student's *t* test was used: ****=p=<0.0001. **e** Representative images from **d** (scale bar: 5 µm). **f** A549 cells were treated with SETX siRNA or mock and exposed to 21% O_2 (Norm) or <0.1% O_2 (Hyp) for 24 hours. The percentage of cells undergoing apoptosis as determined by DAPI staining is shown, Data from three independent experiments (n=3). One-way ANOVA Tukey's multiple comparisons test was used: ***=p=0.0001.Mean ± SEM are displayed.

Supplementary Figure 5

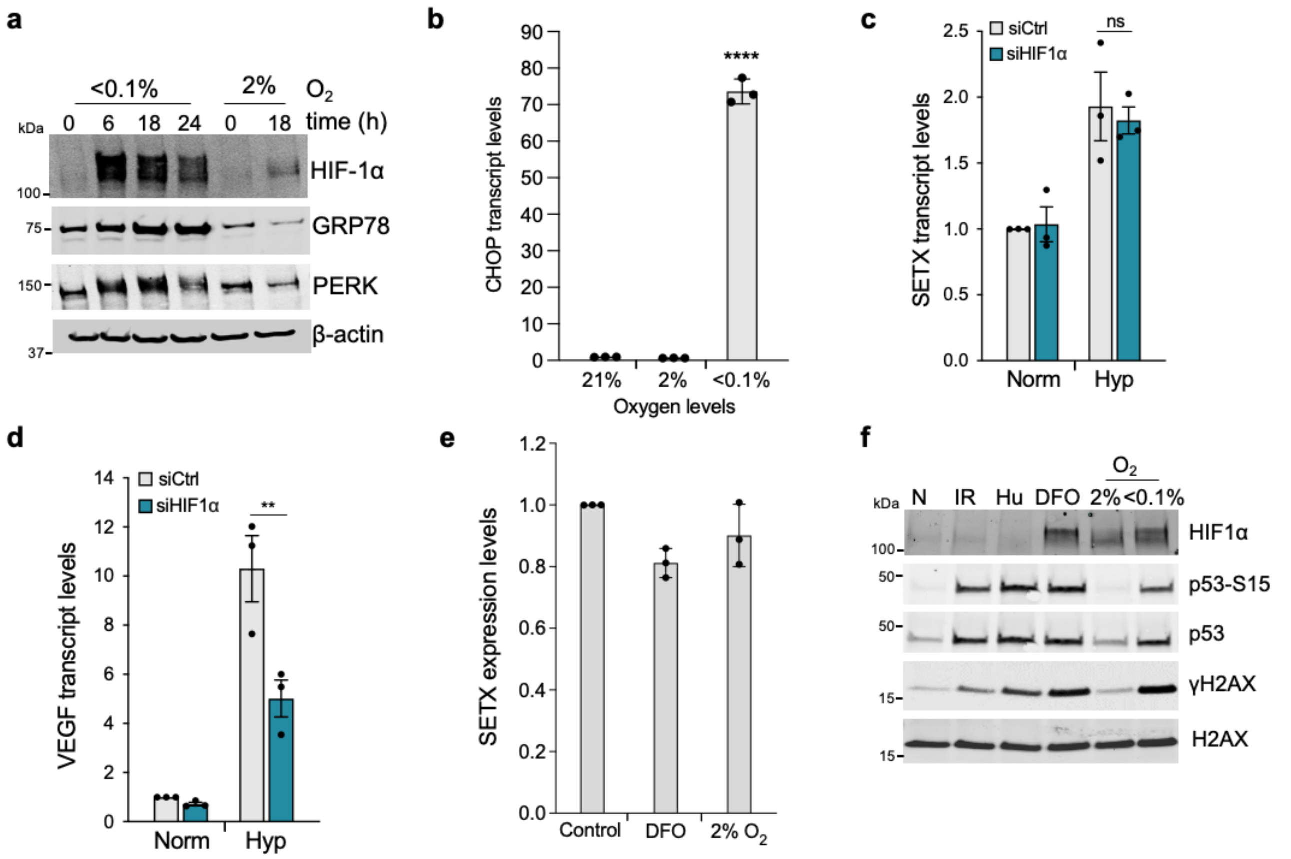

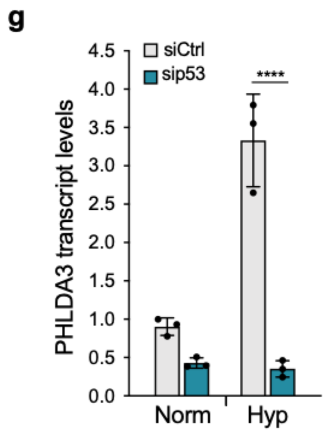

Supplementary Figure 5. **Hypoxia-induced SETX is independent of HIF1α or p53**

a A549 cells were exposed to the oxygen concentrations shown for the times indicated followed by western blotting. Activation of PERK (electrophoretic mobility shift) and induction of GRP78 are indicative of activation of ATF3/4/5. **b** A549 cells were exposed to normoxia (21% O_2) and hypoxia (2% O_2 or < 0.1% O_2) for 16 hours, and CHOP mRNA levels were determined by RT-qPCR. Representative of 3 independent experiments. One-way ANOVA Dunnett's multiple comparisons test was used: ****=p<0.0001. **c** A549 cells were treated with either control siRNA (grey) or HIF1α siRNA (green) and exposed to 21% O_2 (Norm) or <0.1% O_2 (Hyp) for 16 hours. SETX mRNA levels were determined by RT-qPCR. ns = non-significant, one-way ANOVA Tukey's multiple comparisons test was used: ns=p=0.9592. **d** A549 cells were treated with either control siRNA (grey) or HIF1α siRNA (green) and exposed to 21% O_2 (Norm) or <0.1% O_2 (Hyp) for 16 hours. VEGF mRNA levels were determined by RT-qPCR. One-way ANOVA Tukey's multiple comparisons test was used: **=p=0.0056. **e** SETX mRNA levels in non-treated A549 cells (Control), DFO (100 μM) and hypoxia (2% O_2) for 16 hours. Control samples were same as in Figure 5D. **f** Non-treated A549 cells (N), cells exposed to IR (5 Gy) followed by a 1-hour recovery, Hu (1 mM), DFO (100 μM) and hypoxia (2% O_2 or < 0.1% O_2) for 16 hours, were used in western blot assays to determine HIF1α, p53-S15, p53, γH2AX and H2AX protein levels. **g** A549 cells were treated with either control siRNA (grey) or p53 siRNA (green) and exposed to 21 % O2 (Norm) or <0.1% O_2 (Hyp) for 6 hours. PHLDA3 mRNA levels were determined by RT-qPCR. Representative of 3 independent experiments. One-way ANOVA Tukey's multiple comparisons test was used: ****=p<0.0001. (A-G) Data from three independent experiments (n=3), mean ± SEM are displayed unless otherwise indicated.

Supplementary Figure 6

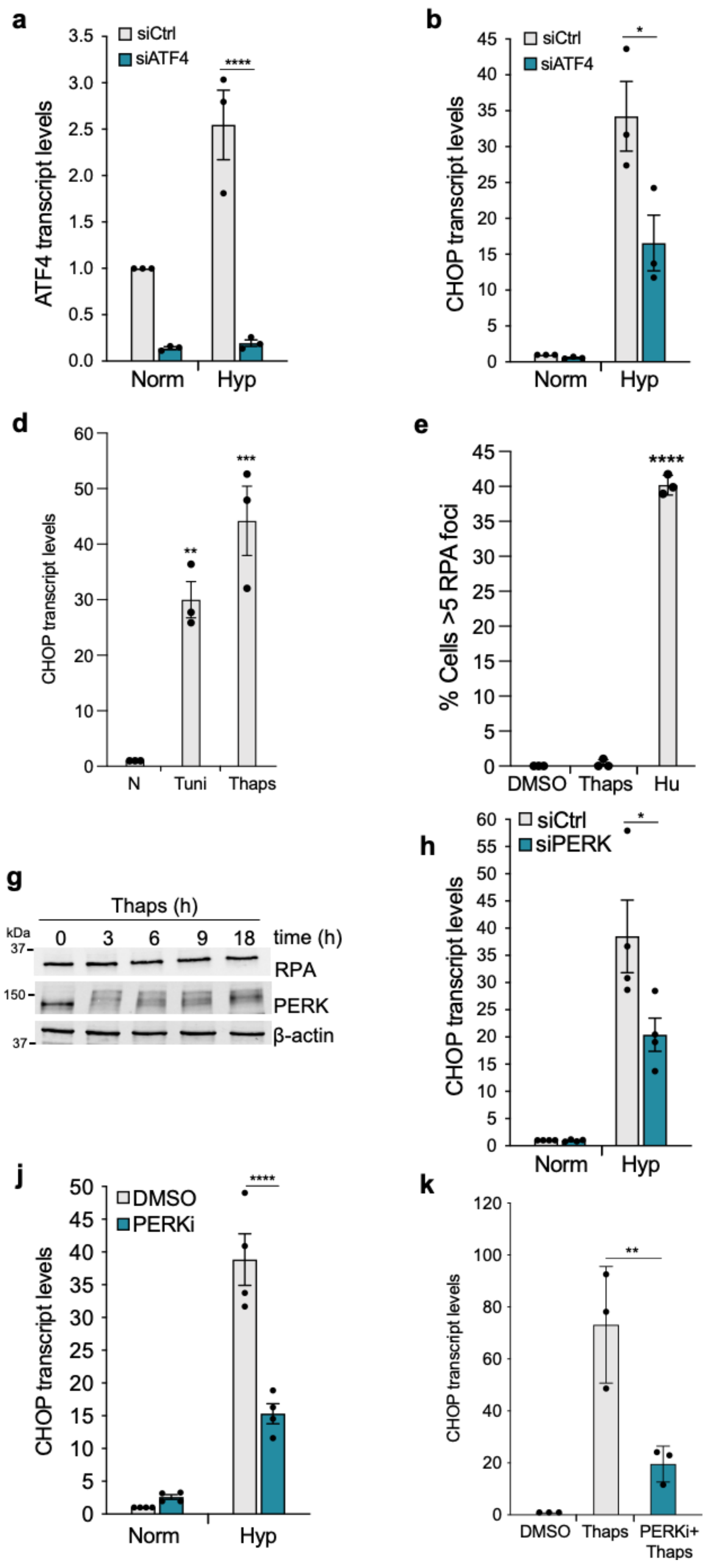

Supplementary Figure 6. **Hypoxia-induced SETX is dependent on PERK/ATF4 signalling**
a A549 cells were treated with either control siRNA (grey) or ATF4 siRNA (green) and exposed to 21% O_2 (Norm) or <0.1% O_2 (Hyp) for 16 hours. ATF4 mRNA levels were determined by RT-qPCR. One-way ANOVA Tukey's multiple comparisons test was used: ****=p<0.0001. **b** A549 cells were treated with either control siRNA (grey) or ATF4 siRNA (green) and exposed to 21% O_2 (Norm) or <0.1% O_2 (Hyp) for 16 hours. CHOP mRNA levels were determined by RT-qPCR. One-way ANOVA Tukey's multiple comparisons test was used: *=p=0.0162. **c** A549 cells were exposed to 21% O_2 (Norm) or <0.1% O_2 (Hyp) for 4 hours, and ChIP-qPCR experiments were performed to determine fold enrichment of ATF4 at the CHOP promoter relative to IgG control. One-way ANOVA Tukey's multiple comparisons test was used: **=p=0.0071. **d** CHOP mRNA levels in non-treated A549 cells (N) and cells exposed to tunicamycin (Tuni, 5 μg/mL) or thapsigargin (Thaps, 2 μM) for 16 hours. One-way ANOVA Dunnett's multiple comparisons test was used: **=p=0.0042, ***=p=0.0005. **e** A549 cells were exposed to Thaps (2 μM, 24 h) or Hu (2 mM, 8 h) and then stained for RPA. % of cells with more than 5 RPA foci are quantified. One-way ANOVA Dunnett's multiple comparisons test was used: ****=p<0.0001. **f** Representative images from part **e** (scale bar: 5 μm). **g** A549 cells were exposed to Thaps (2 μM) for the times indicated followed by western blotting for RPA and PERK. Electrophoretic mobility shift of PERK is indicative of its activation. **h** A549 cells were treated with either control siRNA (grey) or PERK siRNA (green) and exposed to 21% O_2 (Norm) or <0.1% O_2 (Hyp) for 16 hours. CHOP mRNA levels were determined by RT-qPCR. One-way ANOVA Tukey's multiple comparisons test was used: *=p=0.0206. **i** A549 cells were treated with either DMSO or PERKi (10 mM) and exposed to 21% O_2 (Norm) or <0.1% O_2 (Hyp) for 6 hours. SETX mRNA levels were determined by RT-qPCR. One-way ANOVA Tukey's multiple comparisons test was used: **=p=0.0028. **j** A549 cells were treated with either DMSO (grey) or PERK inhibitor (PERKi, 10 μM, green) and exposed to 21% O_2 (Norm) or <0.1% O_2 (Hyp) for 6 hours. CHOP mRNA levels were determined by RT-qPCR. One-way ANOVA Tukey's multiple comparisons test was used: ****=p<0.0001. **k** A549 cells were pre-treated with or without PERKi (10 μM) and exposed to DMSO or Thaps (2 μM) for 6 hours. CHOP mRNA levels were determined by RT-qPCR. One-way ANOVA Tukey's multiple comparisons test was used: **=p=0.0069.
(**a**-**k**) Data from three independent experiments (n=3), mean ± SEM are displayed.

Supplementary Figure 7

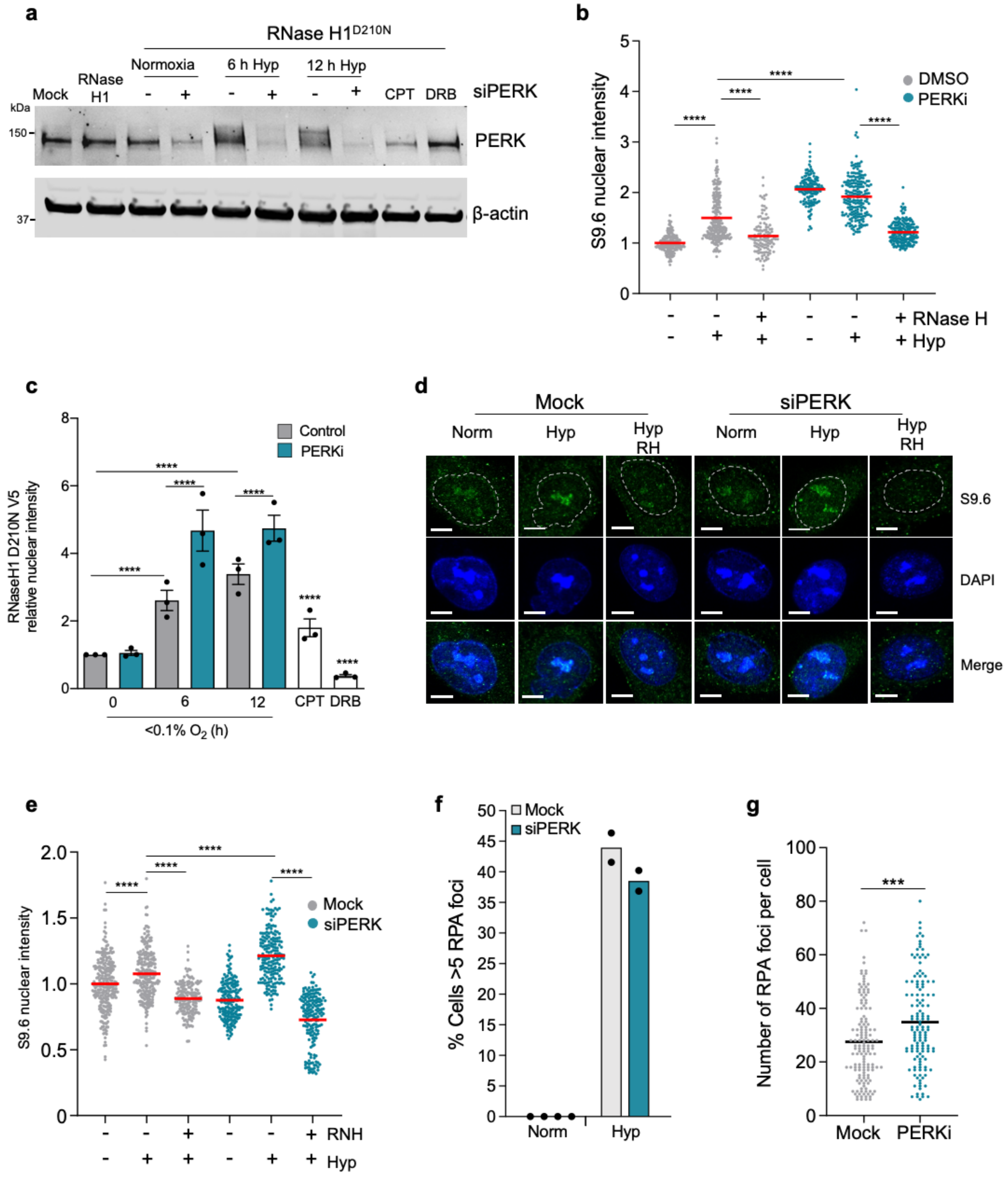

Supplementary Figure 7. **Loss of PERK leads to an accumulation of R-loops and replication stress**
a A549 cells were mock treated, transfected with RNase H1 or transfected with RNase $H1^{D210N}V5$. The RNase $H1^{D210N}V5$ transfected cells were either mock transfected or transfected with PERK siRNA, exposed to 21% O_2 or <0.1% O_2, CPT (10 μM, 20 minutes) or DRB (100 μM, 1 hour). Western blotting for PERK confirms its depletion. Electrophoretic mobility shift of PERK is indicative of its activation. To support data in Fig. 6c, n=3
b A549 cells that were treated with DMSO (grey) or 10 mM PERKi (green) were exposed to 21% O_2 or <0.1% O_2 for 12 hours. Cells were fixed and stained with the S9.6 antibody and DAPI. Where indicated, coverslips were treated with RNase H prior to staining. Data from two independent experiments (n=2) where each dot represents a cell and a minimum of 100 cells were imaged per treatment. One-way ANOVA Tukey's multiple comparisons test was used: ****=p<0.0001.
c A549 cells transfected with RNase $HI^{D210N}V5$ were treated with DMSO (grey) or PERKi (10 μM, green) and exposed to hypoxia (<0.1% O_2), or treated with CPT (10 μM, 20 minutes) or DRB (100 μM, 1 hour). Staining for V5 was then carried out and the nuclear intensity determined. Data from three independent experiments (n=3) where a minimum of 100 cells were imaged per treatment. The two-tailed Student's *t* test was used: ****=p<0.0001.
d A549 cells that were either mock transfected or transfected with PERK siRNA, were exposed to 21% O_2 or <0.1% O_2 for 6 hours. Cells were fixed and stained with the S9.6 antibody and DAPI. Where indicated, coverslips were treated with RNase H prior to staining. Representative images are shown, dashed white line indicates nuclear outline based on DAPI stain that was used to measure the nuclear S9.6 intensity (scale bar: 5 μm).
e Quantification of S9.6 staining from part **d**. Data from two independent experiments (n=2) where each dot represents a cell and a minimum of 100 cells were imaged per treatment. One-way ANOVA Tukey's multiple comparisons test was used: ****=p<0.0001.
f A549 cells with either mock (grey) treatment or PERK siRNA (green) were exposed to hypoxia (<0.1% O_2) for 6 hours, cells were fixed and stained for RPA by immunofluorescence assays. Percentage of cells with greater than 5 RPA foci was quantified. Data from two independent experiments (n=2).
g A549 cells treated with DMSO (grey) or PERKi (10 μM, green), were exposed to hypoxia (<0.1% O_2) for 6 hours, cells were fixed and stained for RPA by immunofluorescence assays. The number of RPA foci per cell was quantified. Data from two independent experiments (n=2) where each dot represents a cell and a minimum of 100 cells were imaged per treatment. Two-tailed Mann-Whitney U test was used: **=p<0.0024.

SI REFERENCES

1 Krieg, A. J. *et al.* Regulation of the histone demethylase JMJD1A by hypoxia-inducible factor 1 alpha enhances hypoxic gene expression and tumor growth. *Molecular and cellular biology* **30**, 344-353, doi:10.1128/MCB.00444-09 (2010).
2 Skourti-Stathaki, K., Proudfoot, N. J. & Gromak, N. Human senataxin resolves RNA/DNA hybrids formed at transcriptional pause sites to promote Xrn2-dependent termination. *Molecular cell* **42**, 794-805, doi:10.1016/j.molcel.2011.04.026 (2011).
3 Wu, H., Sun, H., Liang, X., Lima, W. F. & Crooke, S. T. Human RNase H1 is associated with protein P32 and is involved in mitochondrial pre-rRNA processing. *PloS one* **8**, e71006, doi:10.1371/journal.pone.0071006 (2013).
4 Pizzi, S. *et al.* Reduction of hRNase H2 activity in Aicardi-Goutieres syndrome cells leads to replication stress and genome instability. *Human molecular genetics* **24**, 649-658, doi:10.1093/hmg/ddu485 (2015).
5 Spandidos, A., Wang, X., Wang, H. & Seed, B. PrimerBank: a resource of human and mouse PCR primer pairs for gene expression detection and quantification. *Nucleic acids research* **38**, D792-799, doi:10.1093/nar/gkp1005 (2010).
6 Ye, J. *et al.* Primer-BLAST: a tool to design target-specific primers for polymerase chain reaction. *BMC Bioinformatics* **13**, 134, doi:10.1186/1471-2105-13-134 (2012).
7 Rouschop, K. M. *et al.* The unfolded protein response protects human tumor cells during hypoxia through regulation of the autophagy genes MAP1LC3B and ATG5. *The Journal of clinical investigation* **120**, 127-141, doi:10.1172/JCI40027 (2010).
8 Dobrynin, G. *et al.* KDM4A regulates HIF-1 levels through H3K9me3. *Scientific reports* **7**, 11094, doi:10.1038/s41598-017-11658-3 (2017).
9 Zong, Z. H. *et al.* Implication of Nrf2 and ATF4 in differential induction of CHOP by proteasome inhibition in thyroid cancer cells. *Biochim Biophys Acta* **1823**, 1395-1404, doi:10.1016/j.bbamcr.2012.06.001 (2012).
10 Leszczynska, K. B. *et al.* Hypoxia-induced p53 modulates both apoptosis and radiosensitivity via AKT. *The Journal of clinical investigation* **125**, 2385-2398, doi:10.1172/jci80402 (2015).
11 Schwab, R. A. *et al.* The Fanconi Anemia Pathway Maintains Genome Stability by Coordinating Replication and Transcription. *Molecular cell* **60**, 351-361, doi:10.1016/j.molcel.2015.09.012 (2015).
12 Dhapola, P. & Chowdhury, S. QuadBase2: web server for multiplexed guanine quadruplex mining and visualization. *Nucleic acids research* **44**, W277-283, doi:10.1093/nar/gkw425 (2016).
13 Durinck, S., Spellman, P. T., Birney, E. & Huber, W. Mapping identifiers for the integration of genomic datasets with the R/Bioconductor package biomaRt. *Nat Protoc* **4**, 1184-1191, doi:10.1038/nprot.2009.97 (2009).
14 Buffa, F. M., Harris, A. L., West, C. M. & Miller, C. J. Large meta-analysis of multiple cancers reveals a common, compact and highly prognostic hypoxia metagene. *British journal of cancer* **102**, 428-435, doi:10.1038/sj.bjc.6605450 (2010).
