# Supplementary material for: Hypoxia-induced SETX links replication stress with the unfolded protein response
Source: Nat Commun. 2021 Jun 17;12:3686. doi: 10.1038/s41467-021-24066-z (PMC8211819; doi:10.1038/s41467-021-24066-z)
Supplement: Supplementary file 3 — Reporting Summary [file 41467_2021_24066_MOESM3_ESM.pdf]

nature research

Corresponding author(s): *Double-blind peer review submissions: write DBPR and your manuscript number here instead of author names.*

Last updated by author(s): *YYYY-MM-DD*

Reporting Summary

Nature Research wishes to improve the reproducibility of the work that we publish. This form provides structure for consistency and transparency in reporting. For further information on Nature Research policies, see our Editorial Policies and the Editorial Policy Checklist.

Statistics

For all statistical analyses, confirm that the following items are present in the figure legend, table legend, main text, or Methods section.

n/a	Confirmed	
☐	☒	The exact sample size (*n*) for each experimental group/condition, given as a discrete number and unit of measurement
☐	☒	A statement on whether measurements were taken from distinct samples or whether the same sample was measured repeatedly
☐	☒	The statistical test(s) used AND whether they are one- or two-sided<br>*Only common tests should be described solely by name; describe more complex techniques in the Methods section.*
☐	☒	A description of all covariates tested
☐	☒	A description of any assumptions or corrections, such as tests of normality and adjustment for multiple comparisons
☒	☐	A full description of the statistical parameters including central tendency (e.g. means) or other basic estimates (e.g. regression coefficient) AND variation (e.g. standard deviation) or associated estimates of uncertainty (e.g. confidence intervals)
☒	☐	For null hypothesis testing, the test statistic (e.g. *F*, *t*, *r*) with confidence intervals, effect sizes, degrees of freedom and *P* value noted<br>*Give P values as exact values whenever suitable.*
☒	☐	For Bayesian analysis, information on the choice of priors and Markov chain Monte Carlo settings
☒	☐	For hierarchical and complex designs, identification of the appropriate level for tests and full reporting of outcomes
☒	☐	Estimates of effect sizes (e.g. Cohen's *d*, Pearson's *r*), indicating how they were calculated

Our web collection on statistics for biologists contains articles on many of the points above.

Software and code

Policy information about availability of computer code

Data collection	No code was used
Data analysis	The 5'EU, S9.6 and V5 mean nuclear intensity signal was determined using ImageJ plugin/algorithm kindly provided by Dr Kienan Savage, Queen's University Belfast (Vohhodina et al., NAR 2017)

For manuscripts utilizing custom algorithms or software that are central to the research but not yet described in published literature, software must be made available to editors and reviewers. We strongly encourage code deposition in a community repository (e.g. GitHub). See the Nature Research guidelines for submitting code & software for further information.

Data

Policy information about availability of data

All manuscripts must include a data availability statement. This statement should provide the following information, where applicable:

- Accession codes, unique identifiers, or web links for publicly available datasets
- A list of figures that have associated raw data
- A description of any restrictions on data availability

RNA-seq is at GSE157371
Figures 3B, and S3 and S4 have associated raw data (RNA-seq)
RNA-seq is available post publication

Field-specific reporting

Please select the one below that is the best fit for your research. If you are not sure, read the appropriate sections before making your selection.

☒ Life sciences ☐ Behavioural & social sciences ☐ Ecological, evolutionary & environmental sciences

For a reference copy of the document with all sections, see nature.com/documents/nr-reporting-summary-flat.pdf

Life sciences study design

All studies must disclose on these points even when the disclosure is negative.

Sample size	No sample sizes were pre-determined statistically. For DNA Fibre assays, a minimum of 80 fibres were measured for each treatment. For immunofluorescence microscopy at least 100 cells were measured for each treatment.
Data exclusions	No data was excluded
Replication	All findings were replicated and reproduced
Randomization	Not relevant
Blinding	For immunofluorescence based studies the person analyzing the data was blinded to which sample was which.

Reporting for specific materials, systems and methods

We require information from authors about some types of materials, experimental systems and methods used in many studies. Here, indicate whether each material, system or method listed is relevant to your study. If you are not sure if a list item applies to your research, read the appropriate section before selecting a response.

Materials & experimental systems

n/a	Involved in the study	
☐	☒	Antibodies
☐	☒	Eukaryotic cell lines
☒	☐	Palaeontology and archaeology
☒	☐	Animals and other organisms
☒	☐	Human research participants
☒	☐	Clinical data
☒	☐	Dual use research of concern

Methods

n/a	Involved in the study	
☒	☐	ChIP-seq
☒	☐	Flow cytometry
☒	☐	MRI-based neuroimaging

Antibodies

Antibodies used	SETX (Bethyl A301-105), KAP1 (Bethyl A300-274A), GRP78 (BD Biosciences 610978), HIF1a (BD Biosciences 610958), ATM-S1981 (Abcam Ab81292), ATM (Cell Signaling 2873), RPA (Cell Signaling 2208), KAP1-S824 (Cell Signaling 4127), p53-S15 (Cell Signaling 9284), p53 (Santa Cruz sc-126), B-actin (Santa Cruz sc-69879), gH2AX (Millipore 05-636), H2AX (Millipore 07-627), RNase H1 (Santa Cruz sc-365267), Chk1 (Santa Cruz sc-8408), H3K9me2 (Cell Signaling 9753), H3k4me2 (Cell Signaling 9725), H3K9Ac (Cell Signaling 9671), H3-S10 (Cell Signaling 9701), H3K36me3 (Cell Signaling 4909), H3 (Cell Signaling 4499), Chk1-S317 (Cell Siginaling 2344), PERK (Cell Signaling 3192), H3K9me3 (Millipore 07-523), RPA-S4/S8 (Abcam Ab243866), B-Tubulin (Abcam Ab179513)
Validation	All antibodies used in this study were commercially available and validated by manufacturer. In addition, positive and negative controls were used in our experiments to demonstrate that the observed signal on a western blot reacted as expected. Bands were all also checked for appropriate size.

Eukaryotic cell lines

Policy information about cell lines

Cell line source(s)	All cells lines (RKO, HCT116 and A549) were obtained from the ATCC
Authentication	Cell lines are periodically checked by STR profiling. Fresh cells are thawed and used every 3-6 months to reduce small risk of cell lines having become contaminated with other lines.
Mycoplasma contamination	Cells lines are regularly tested for mycoplasma (HEK-Blue PlasmoTest Detection kit, InvivoGen)

April 2020

Commonly misidentified lines<br>(See ICLAC register)	Not relevant
